# Impact of coronavirus 2019 on mental health and lifestyle adaptations of pregnant women in the United Arab Emirates: a cross-sectional study

**DOI:** 10.1186/s12884-021-03941-z

**Published:** 2021-07-19

**Authors:** Mona Hashim, Ayla Coussa, Ayesha S. Al Dhaheri, Amina Al Marzouqi, Samer Cheaib, Anastasia Salame, Dima O. Abu Jamous, Farah Naja, Hayder Hasan, Lily Stojanovska, Maysm N. Mohamad, Mo’ath F. Bataineh, MoezAlIslam E. Faris, Rameez Al Daour, Reyad S. Obaid, Sheima T. Saleh, Tareq M. Osaili, Leila Cheikh Ismail

**Affiliations:** 1 Department of Clinical Nutrition and Dietetics, College of Health Sciences, University of Sharjah, Sharjah, 27272 United Arab Emirates; 2 Nutrition and Dietetics Program, School of Health Sciences, Universiti Sains Malaysia, 16150 Kubang Kerian, Kelantan Malaysia; 3 Warwick Medical School, University of Warwick, Coventry, CV4 7AL UK; 4 Department of Nutrition and Health, College of Medicine and Health Sciences, United Arab Emirates University, Al Ain, 15551 United Arab Emirates; 5 Department of Health Services Administration, College of Health Sciences, Research Institute of Medical and Health Sciences (RIMHS), University of Sharjah, Sharjah, 27272 United Arab Emirates; 6 Fakih IVF Clinics, Fetal Medicine Department, Jumeirah 1, Dubai, 72960 United Arab Emirates; 7 Fakih IVF Clinics, Obstetrics and Gynecology Department, Ashraj 13, Al Ain, 31453 Abu Dhabi, United Arab Emirates; 8 Research Institute for Medical and Health Sciences, University of Sharjah, Sharjah, 27272 United Arab Emirates; 9 Victoria University, Institute for Health and Sport, Melbourne, 14428 Australia; 10 Department of Sport Rehabilitation, Faculty of Physical Education and Sport Sciences, The Hashemite University, Zarqa, Jordan; 11 Department of Nutrition and Food Technology, Faculty of Agriculture, Jordan University of Science and Technology, Irbid, 22110 Jordan; 12 Nuffield Department of Women’s & Reproductive Health, University of Oxford, Oxford, OX1 2JD UK

**Keywords:** COVID-19 pandemic, Mental health, Psychological factors, Pregnant women

## Abstract

**Background:**

In light of the pandemic, pregnant women are particularly vulnerable to increased psychological distress and in need of imperative preventive measures. This study aimed to investigate the impact of the pandemic on mental health, lifestyle adaptations, and their determinants among pregnant women in the United Arab Emirates.

**Methods:**

A survey was conducted electronically between June and August 2020. Pregnant women were recruited from prenatal clinics in the UAE and invited to participate in an online survey developed on Google Forms. The questionnaire included socio-demographic characteristics, the Impact of Event Scale- Revised, the Perceived Support Scale and lifestyle-related factors.

**Results:**

A total of 384 pregnant women completed the questionnaire of whom 20.6% were in their 1st trimester, 46.1% in their 2nd and 33.3% in their 3rd trimester. The mean IES-R score for the respondents was 26.15 ± 13.55, corresponding to a mild stressful impact, which did not differ significantly among trimesters of pregnancy. Pregnant women expressed increased stress from staying home (64%), work (40%), feeling frightened (66%) and apprehensive (59%). Women reported increased support and sharing their feelings with family members (59%), mainly in the 1st and 3rd trimester of pregnancy (P < 0.05). There was a greater attention to mental health (48%), resting time (55.3%), and relaxing time (57.3%); while a decreased amount of time was spent engaging in physical activities (53.6%), which differed significantly between trimesters (P = 0.02).

**Conclusions:**

The COVID-19 pandemic was associated with a mild stressful impact among pregnant women in the UAE, braced by strong family support and self-care mental health behaviors.

## Background

The viral disease emerged in the city of Wuhan (China) in late December 2019 and was later identified as the novel coronavirus (COVID-19) [1]. The world witnessed an exponential surge of cases, and it was declared a global pandemic by the World Health Organization (WHO) in March 2020 [2]. As of 28 February 2021, more than 113 million cases had been recorded globally with more than 2.5 million deaths, and over 388,594 cases were confirmed in the United Arab Emirates (UAE) with a total of 1213 deaths [3].

Serious preventive and precautionary strategies were implemented in response to the alarming spread of a severe acute respiratory syndrome coronavirus 2 infection, and these included border closures, suspension of flights, complete and partial lockdowns, quarantine, physical distancing, and strict hygiene measures [4]. Despite global efforts in the development of various treatments including vaccines for this novel virus, the unpredictability and uncertainty of the pandemic have created psychological distress and anxiety in the general population [5, 6]. Additionally, the pandemic has led to increased unemployment and impaired financial status [7, 8]. Consequently, the pandemic has resulted in a moderate to severe adverse impact on the mental health of the general population and particularly in women as reported in some European countries and China [9, 10].

Pregnancy represents a period with profound physiological changes and a stressor mechanism on the inflammatory system [11]. Due to the weakened immune system, pregnant women are classified among the most vulnerable group to contract communicable diseases, such as COVID-19 [12]. Pregnant women are also at increased risk of severe illnesses from the virus and adverse pregnancy-related outcomes (e.g. preterm birth) [13]. In addition, pregnancy may be associated with psychological distress (such as anxiety), related but not limited to placental hormones, which are considered stress triggers [14, 15]. As pregnancy progresses, these hormone levels increase exponentially, which may explain trimester-dependent changes in the mental well-being of pregnant women [16].

In March 2020, lockdown regulations were implemented in the UAE to contain the spread of the virus and resulted in a limited provision of face-to-face medical services. Medical visits and routine follow-ups were restricted to emergency and serious cases during the lockdown; this may have caused some anxiety among pregnant women because they could not adequately follow up on the health of their fetus and could have impaired their overall mental well-being [17]. In addition, some pregnant women might not have been attending their routine prenatal visits, being fearful of catching the virus [18]. Previous studies have assessed the early impacts of COVID-19 on psychological stress and anxiety among different populations, including in China, Italy, and Iran [6, 15, 19, 20]. To our knowledge, this is the first study that has investigated these consequences among pregnant women in the UAE, and their lifestyle adaptations in response to the pandemic. In the current study, we assessed the early impact of the pandemic on mental health and lifestyle adaptations among pregnant women living in the UAE and compared them across the three trimesters of pregnancy. We also aimed to identify significant determinants of negative mental health well-being.

## Methods

### Subjects

A cross-sectional study was conducted in the UAE amid the pandemic of COVID-19 between June and August 2020. Pregnant women were recruited from three prenatal clinics in the emirates of Abu Dhabi, Dubai, and Sharjah. These clinics are the biggest providers of antenatal care in the UAE and were visited by pregnant women of various nationalities (both locals and expats) living in the UAE. Eligible participants were pregnant women ≥18 years residing in any of the seven emirates of the UAE (Abu Dhabi, Dubai, Sharjah, Ajman, Um Al Quwain, Ras Al Khaima, Fujairah). The study protocol was approved by the Research Ethics Committee at the University of Sharjah (REC-20-06-09-01).

### Sample size calculation

The number of respondents required in this study, and the approximate effect size estimated at 19%, was based on a recent large cohort study that reported a 19% prevalence of mental health disorders in pregnancy [21] that was calculated as follows:

$$ \mathrm{N}=\mathrm{Z}2\ \mathrm{x}\ \mathrm{P}\ \mathrm{x}\ \left(1-\mathrm{P}\right)/\mathrm{e}2 $$

$$ N=1.962\ \mathrm{x}\ 0.19\ \mathrm{x}\ \left(1-0.19\right)/0.052 $$

$$ N=236 $$

Where: z = standard normal deviation set at 95% confidence level (1.96), *p* = prevalence of mental health disorder in pregnancy. Assuming incomplete submission of the survey at a rate of 10%, the research team aimed to recruit 260 respondents.

### Questionnaires

The questionnaires used in the data collection were adapted from the literature and reviewed by a panel of experts in the field including a mental health researcher, an antenatal epidemiologist, and an epidemiologist. The questionnaires were first developed in English and were later translated into Arabic following internationally accepted methodology [22]. Both the English and Arabic forms were compared and verified for parallel form reliability. The questionnaires were later formatted using Google Forms. Prior to launching the survey, the questionnaires were pilot tested on a sample of 10 pregnant women, and the questions were checked for clarity and cultural appropriateness. The data from the pilot testing was not included in the analysis in this study. Women were asked to give consent before starting the survey and data were collected anonymously. There were no incentives for participating in completing the questionnaire. The questionnaire consisted of five parts. The first part addressed the sociodemographic characteristics: age, the emirate of residence, education level, employment, work location, and pregnancy trimester. The second part included the Impact of Event Scale-Revised (IES-R), an easily administered self-report questionnaire that contains 22 items and was designed as a measure of post-traumatic stress disorder (PTSD) symptoms [23, 24]. The IES-R was validated in the Arabic language [25] and was also modified and validated to assess the psychological impact of the COVID-19 outbreak (IES-COVID-19) [26]. Respondents were asked to rate the items based on how relevant they were to them in the past 7 days. The response for each question was scored based on a five-point Likert scale ranging from 0 (not at all) to 4 (extremely), generating a total score (range from 0 to 88), with higher scores indicating a higher psychological impact. The total IES-R score was divided into four categories regarding the psychological impact: normal (0 to 23), mild (24 to 32), moderate (33 to 36), and severe (≥ 37) [27]. The third part of the questionnaire focused on negative indicators of mental health, whereby respondents were asked if they were experiencing increased stress from work, financial status, and/or from staying at home during the pandemic. The questions were adapted from a previous study conducted among pregnant women in China [20]. The fourth part of the questionnaire revolved around the perception of family and/or friends’ support and the extent to which they had shared their feelings in the past month during the pandemic. The questions were adapted from the Perceived Support Scale (PSS) with modifications [28]. The response options to these questions were: much increased, increased, same as before, decreased, and much decreased. The last part of the questionnaire covered lifestyle adaptations during the pandemic (such as time spent to rest, exercise and eating pattern, as well as use of herbal supplements such as turmeric, ginger, vitamin C) and immunity-boosting strategies using the Mental Health Lifestyle Scale (MHLSS) [28].

### Statistical analyses

Statistical analyses were performed using the Statistical Package for the Social Sciences (SPSS) version 26.0 (IBM, Chicago, IL, USA). The Shapiro-Wilk test was used to check data normality. Descriptive statistics for the sociodemographic characteristics were reported as frequencies and percentages. The continuous values of the IES-R score were presented as means and standard deviations (SD), and an ANOVA test was used to determine whether there were any differences in the IES-R scores between the trimesters of pregnancy. Associations and comparisons among different categorical variables (negative mental health indicators, support from family and friends indicators, and mental health-related lifestyle adaptation indicators) were determined with the Chi-square test, within trimesters of pregnancy. Univariate and multivariate logistic regression tests were used to evaluate the predictors of the IES-R score category in pregnancy and were adjusted for employment. Given the small number of responders in some variables, the following answers were merged into one category: “much increased” and “increased”, and “decreased” and “much decreased”. Results were significant for *P*-value ≤ 0.05 with a 95% confidence interval (CI).

## Results

### 1. Respondents’ characteristics

The questionnaire was completed by 384 pregnant women. The sociodemographic characteristics of respondents are presented in Table 1. Two-thirds of the study population (67.4%) comprised individuals aged 26–35 years. About half of the pregnant women (46.1%) were in their 2nd trimester, a third (33.3%) in their 3rd, and the remaining (20.6%) were in their 1st trimester. The vast majority of the respondents were highly educated (79.7%), with more than one-third of them having either a diploma (38.8%) or a university degree (40.9%), and 64% were employed, of whom 53.6% were working remotely from home.

**Table 1:** Sociodemographic characteristics of pregnant women (*N* = 384)

Sociodemographic variables		% (n)
UAE Residency	Abu Dhabi	39.1 (150)
	Dubai	45.1 (173)
	Others	17.8 (61)
Age (Year)	18–25	7.6 (29)
	26–35	67.4 (259)
	36–45	25 (96)
Trimester into pregnancy	1–12 weeks	20.6 (79)
	13–26 weeks	46.1 (177)
	≥27 weeks	33.3 (128)
Education	High school	20.4 (78)
	College/Diploma	38.8 (149)
	University Degree	40.9 (157)
Employment	Yes	64 (246)
	No	36 (138)
Work/study from home	Yes	53.6 (206)
	No	46.4 (178)

### 2. Impact of event scale-revised and indicators of negative mental health

The IES-R scores by trimester of pregnancy are presented in Table 2. Overall, the mean IES-R score was 26.15 ± 13.55 for the whole sample and did not differ significantly between trimesters of pregnancy (*P* = 0.06). In relation to IES-R categorical distribution, almost half of the respondents (47.1%) fell within the normal IES-R score range (0–23). There was no significant difference in IES-R categories distribution between trimesters of pregnancy (*P* = 0.12).

**Table 2:** Difference in Impact Event Scale-Revised (IES-R) scores between trimesters of pregnancy

Event Scale-Revised (IES-R) Score	All (*n* = 384)	Pregnancy trimesters			*P-value*
		1^st^ Trimester (*n* = 79)	2^nd^ Trimester (*n* = 177)	3^rd^ Trimester (*n* = 128)	
IES-R, mean ± SD					
Total score	26.15 ± 13.55	24.99 ± 14.28	24.98 ± 12.21	28.48 ± 14.61	^a^0.06
IES-R category %					
Normal (0–23)	47.1	45.6	50.8	43.0	^b^0.12
Mild (24–32)	17.7	15.2	18.6	18.0	
Moderate (33–36)	12.2	16.4	13.0	8.6	
Severe (≥37)	23.0	22.8	17.6	30.4	

*SD* Standard Deviation; *P* < 0.05 vs. significance for IES-R scores by ^a^ ANOVA; ^b^ IES-R scores categories by Chi-square test.

The indicators of negative mental health by trimester of pregnancy are shown in Table 3. Work-related stress was equally unchanged (37.2%) or increased (39%) during the pandemic, which was also the case for increased stress from the financial situation (45.1 and 34.1% respectively). Moreover, about two-thirds of the respondents reported increased stress from staying at home (63.5%) and experiencing increased fear (66.1%), apprehension (58.6%), and helplessness (44.5%) due to the COVID-19 pandemic. When comparing negative mental health indicators by trimester of pregnancy, increased financial stress and feelings of fear, apprehension, and helplessness differed significantly between trimesters (*P =* 0.009*; P =* 0.05*; P =* 0.04*;* and *P =* 0.02 respectively)*.* Women who experienced the highest prevalence of negative mental health were in their 3rd trimester of pregnancy.

**Table 3:** Differences in negative mental health indicators during the COVID-19 pandemic between trimesters of pregnancy

Indicators	All *(N* = 384)	Pregnancy trimesters			
		1^st^ Trimester *(n* = 79)	2^nd^ Trimester (*n* = 177)	3^rd^ Trimester (*n* = 128)	*P-* value
Increased stress from work % (n)					
Decreased	23.8 (91)	16.5 (13)	25.4 (45)	25.8 (33)	0.73
Same as before	37.2 (143)	38.0 (30)	36.7 (65)	37.5 (48)	
Increased	39.0 (150)	45.5 (36)	37.9 (67)	36.7 (47)	
Increased financial stress % (n)					
Decreased	20.8 (80)	19.0 (15)	22.6 (40)	19.5 (25)	0.009
Same as before	45.1 (173)	58.2 (46)	47.5 (84)	33.6 (43)	
Increased	34.1 (131)	22.8 (18)	29.9 (53)	46.9 (60)	
Increased stress from staying at home % (n)					
Decreased	13.8 (53)	12.7 (10)	14.7 (26)	13.3 (17)	0.11
Same as before	22.7 (87)	25.3 (20)	23.2 (41)	20.3 (26)	
Increased	63.5 (244)	62.0 (49)	62.1 (110)	66.4 (85)	
Felt frightened due to COVID-19% (n)					
Decreased	14.6 (56)	16.5 (13)	12.4 (22)	16.4 (21)	0.05
Same as before	19.3 (74)	27.8 (22)	18.6 (33)	14.8 (19)	
Increased	66.1 (254)	55.7 (44)	26.0 (46)	68.8 (88)	
Felt apprehensive due to COVID-19					
Decreased	15.6 (60)	22.8 (18)	14.7 (26)	12.5 (16)	0.04
Same as before	25.8 (99)	31.6 (25)	23.2 (41)	25.8 (33)	
Increased	58.6 (225)	45.6 (36)	62.1 (110)	61.7 (79)	
Felt helpless due to COVID-19					
Decreased	22.7 (87)	24.1 (19)	22.6 (40)	21.9 (28)	0.02
Same as before	32.8 (126)	39.2 (31)	33.9 (60)	27.3 (35)	
Increased	44.5 (171)	36.7 (29)	43.5 (77)	50.8 (65)	

*P* < 0.05 by Chi-square test.

Perception of family and social support during the pandemic by trimester of pregnancy is presented in Table 4. Overall, respondents reported increased support and sharing feelings with family (59.4 and 59.1%) respectively. Caring for family members was also increased during the pandemic, as reported by approximately two-thirds of the respondents (61.7%). Nearly half of the respondents (44.8%) reported that the support received from friends as well as “sharing feelings when feeling blue” (47.1%) remained the same during the pandemic. The response to “support from family members”, “sharing feelings with others when feeling anxious” and “caring for family” differed significantly between trimesters of pregnancy (*P* = 0.01, *P* = 0.01, and *P* < 0.001 respectively). Concerning differences between trimesters, respondents in the 1^st^ and 3^rd^ trimesters followed a similar distribution of percentages.

**Table 4:** Differences in the perception of family and social support during the COVID-19 pandemic between trimesters of pregnancy

Family and social support indicators	All *(n* = 384)	Pregnancy trimesters			
		1^st^ Trimester *(n* = 79)	2^nd^ Trimester (*n* = 177)	3^rd^ Trimester (*n* = 128)	*P*-value
Getting support from friends % (n)					
Decreased	21.1 (81)	20.3 (16)	22.0 (39)	20.4 (26)	0.49
Same as before	44.8 (172)	50.6 (40)	45.2 (80)	40.6 (52)	
Increased	34.1 (131)	29.1 (23)	32.8 (58)	39.0 (50)	
Getting support from family members % (n)					
Decreased	8.0 (31)	1.27 (1)	9.0 (16)	10.9 (14)	0.01
Same as before	32.6 (125)	31.6 (25)	36.2 (64)	28.2 (36)	
Increased	59.4 (228)	67.1 (53)	54.8 (97)	60.9 (78)	
Shared feelings with family members % (n)					
Decreased	8.6 (33)	6.3 (5)	9.6 (17)	8.6 (11)	0.12
Same as before	32.3 (124)	27.9 (22)	37.3 (66)	28.1 (36)	
Increased	59.1 (227)	65.8 (52)	53.1 (94)	63.3 (81)	
Shared feelings with others when feeling anxious % (n)					
Decreased	22.4 (86)	24.1 (19)	25.4 (45)	17.2 (22)	0.01
Same as before	47.1 (181)	45.5 (36)	49.7 (88)	44.6 (57)	
Increased	30.5 (117)	30.4 (24)	24.9 (44)	38.2 (49)	
Caring for family members’ feelings % (n)					
Decreased	5.5 (21)	2.5 (2)	7.9 (14)	3.9 (5)	< 0.001
Same as before	23.4 (90)	10.2 (8)	32.8 (58)	18.8 (24)	
Increased	61.7 (273)	87.3 (69)	59.3 (105)	77.3 (99)	

*P* < 0.05 by Chi-square test

### 3. Lifestyle adaptations during the COVID-19 pandemic

Table 5 summarizes the impact of COVID-19 on mental health-related lifestyle changes by trimester of pregnancy. During the pandemic, paying attention to mental health was reported to be equally unchanged (42.4%) or increased (48.2%) compared to pre-pandemic times. Time spent resting (55.2%) and relaxing (57.3%) was increased while exercising was decreased (53.6%). When comparing the three trimesters of pregnancy, only time spent exercising differed significantly between the three trimesters (*P* = 0.02).

**Table 5:** Differences in mental health-related lifestyle adaptations during the COVID-19 pandemic between trimesters of pregnancy

Mental health-related lifestyle adaptation indicators	All *(n* = 384)	Pregnancy trimesters			
		1^st^ Trimester *(n* = 79)	2^nd^ Trimester (*n* = 177)	3^rd^ Trimester (*n* = 128)	*P-* value
Pay attention to mental health % (n)					
Decreased	9.4 (36)	7.6 (6)	11.3 (20)	7.8 (10)	0.11
Same as before	42.4 (163)	38.0 (30)	45.2 (80)	41.4 (53)	
Increased	48.2 (185)	54.4 (43)	43.5 (77)	50.8 (65)	
Time spent to rest % (n)					
Decreased	16.4 (63)	17.7 (14)	15.3 (27)	17.2 (22)	0.74
Same as before	28.4 (109)	29.1 (23)	26.0 (46)	31.3 (40)	
Increased	55.2 (212)	53.2 (42)	58.7 (104)	51.5 (66)	
Time spent to relax % (n)					
Decreased	20.8 (80)	20.3 (16)	20.9 (37)	21.1 (27)	0.99
Same as before	21.9 (84)	24.1 (19)	20.9 (37)	21.9 (28)	
Increased	57.3 (220)	55.6 (44)	58.2 (103)	57.0 (73)	
Time spent to exercise % (n)					
Decreased	53.6 (206)	55.6 (44)	55.4 (98)	50.0 (64)	0.02
Same as before	32.6 (125)	34.3 (27)	31.6 (56)	32.8 (42)	
Increased	13.8 (53)	10.1 (8)	13.0 (23)	17.2 (22)	

*P* < 0.05 by Chi-square test

Perceptions of immunity boosters and eating behaviors pre- and during the COVID-19 pandemic are shown in Table 6. Nearly all (93%) of the respondents reported not taking supplements to boost their immune system during the pandemic. Pregnant women rated consumption of balanced diets as a number one strategy to enhance the immune system, followed by adequate fluids and proper sleep (respectively: 80, 70, and 61%). More than half of the respondents (58.1%) did not perceive physical activity as an immunity booster. In relation to eating behaviors, homemade meals (88%) and fast foods (83%) were the main eating patterns pre-COVID, followed by eating at restaurants (29%). During the pandemic, homemade food was the only dominant eating pattern, while fast food intake dropped to 3.4% and eating at restaurants to 7.6%. The “healthy food” options represented a small percentage in respondents’ eating patterns pre-COVID (14.3%) and dropped further during the pandemic (7.3%).

**Table 6:** Perceptions of immunity boosters and eating behaviors pre- and during the COVID-19 pandemic

Immunity boosters and eating behavior	Yes % (n)	No % (n)
Immunity booster behaviors		
Taking anything to boost the immune system	7.3 (28)	92.7 (356)
Eating balanced diet	79.9 (307)	20.1 (77)
Taking antenatal supplements	45.1 (173)	54.9 (211)
Drinking adequate fluids	69.8 (268)	30.2 (116)
Practicing physical activity	41.9 (161)	58.1 (223)
Consuming herbs and spices	10.7 (41)	89.3 (343)
Getting proper sleep	60.9 (234)	39.1 (150)
Managing/Minimizing stress	54.4 (209)	45.6 (175)
Meals consumed pre-COVID-19		
Homemade	88.0 (338)	12.0 (46)
Frozen ready-to-eat	4.9 (19)	95.1 (365)
Fast food	83 (216)	78.4 (301)
Restaurants	28.9 (111)	71.1 (273)
Healthy food	14.3 (55)	85.7 (329)
Meals consumed during COVID-19		
Homemade	97.9 (376)	2.1 (8)
Frozen ready-to-eat	3.4 (13)	96.6 (371)
Fast food	5.2 (20)	94.8 (364)
Restaurants	7.6 (29)	92.4 (355)
Healthy food	7.3 (28)	92.7 (356)

Predictors of IES-R scores are presented in Table 7. Multiple logistic regression revealed an inverse relationship between the following predicting factors and IES-R scores: family and friends’ support; sharing feelings with family and friends; as well as caring for others (all *P* < 0.01). In addition, taking natural immune-boosting products and antenatal supplements was also significantly associated with lower IES-R scores (*P* < 0.01). When adjusting for employment, family support, sharing feelings with friends, and taking immune boosters remain significant predictors of lower IES-R scores (*P* < 0.01).

**Table 7:** Determinants of negative mental health by logistic regression, adjusting for employment

Determinant of negative mental health	Adjusted analysis			Unadjusted analysis		
	B-value	95% CI	*P-*value	B-value	95% CI	*P-*value
Employment Ref. (Employed)	0.70	1.27, 3.20	0.003	0.71	1.24, 3.31	0.005
Friends’ support Ref. (Increased)	− 0.75	0.30, 0.74	0.001	− 0.09	0.52, 1.59	0.75
Family support Ref. (Increased)	−1.07	0.28, 0.55	< 0.001	− 0.80	0.24, 0.83	0.001
Shared feeling with family Ref. (Increased)	−0.77	0.30, 0.73	0.001	−0.08	0.48, 1.78	0.81
Shared feeling with friends Ref. (Increased)	−0.90	0.26, 0.63	0.001	−0.60	0.32, 0.95	0.03
Caring for family Ref. (Increased)	−0.65	0.32, 0.86	< 0.001	−0.001	0.52, 1.90	0.99
Taking attention of your mental health Ref. (Increased)	0.05	0.51, 2.14	0.90	–	–	–
Practicing physical activity Ref. (No)	0.12	0.74, 1.73	0.57	–	–	–
Age Ref. (25–35 years)	−0.01	0.61, 1.63	0.98	–	–	–
Getting proper sleep Ref. (No)	−0.04	0.63, 1.48	0.87	–	–	–
Consuming herbs Ref. (No)	−0.19	0.43, 1.61	0.58	–	–	–
Managing stress Ref. (No)	−0.07	0.61, 1.42	0.74	–	–	–
Eating balanced diets Ref. (No)	−0.14	0.68, 1.92	0.61	–	–	–
Taking anything to boost the immune system (e.g., turmeric, ginger, vitamin C) Ref. (No)	−0.66	0.34, 0.79	0.002	−0.55	0.36, 0.90	0.01
Taking antenatal supplements Ref. (No)	−0.04	0.43, 0.98	0.05	−0.24	0.49,1.23	0.30

*Ref*. Reference, *CI* confidence interval.

## Discussion

This study is the first to assess mental health, psychological impact, and lifestyle adaptations among pregnant women in the UAE during the COVID-19 pandemic between June and August 2020. Overall, respondents were young, educated women, and half of them were working from home. The COVID-19 outbreak had a mild psychological impact on pregnant women residing in the UAE (IES-R score: 24–32), with no differences among trimesters of pregnancy. The mild psychological impact on our respondents is possibly explained by the reassuring and effective strategies taken by the UAE government to mitigate and control the epidemic. These measures included but were not limited to implementing of complete and partial lockdowns, suspending flights and issuing new UAE visas, closing shopping centers and entertainment locations, suspending prayers in all places of worship, initiating work from home and distance learning, and providing delivery services like delivering medications to chronically ill patients [29, 30].

Unlike the results presented in this study, a moderate-to-severe psychological impact was reported by pregnant women in Italy (mean IES score of 36.9) and in China (mean IES score of 31.4) [15, 20]. The cross-sectional study conducted in Italy reported that women in their 1st trimester of pregnancy were more likely to have an IES-R score > 26 compared to those in their 2nd or 3rd trimesters [15]. In addition, regardless of the trimester of pregnancy, increased stress from staying at home during the pandemic was one of the main negative triggers on mental health in the current study. This is likely explained by the uncertain duration of the lockdown and the consequences of the emerging situation (e.g., fear of losing their jobs, homeschooling, health concerns, and fear of getting infected). Although in the current study the financial stress was rated to be significantly increased by pregnant women in their 3rd trimester, it was considered unchanged for those in their 1^rst^ and 2nd trimesters. Stress from work did not differ between trimesters. Given that the survey was conducted at an early stage in the COVID-19 pandemic, it might have been too soon for respondents to feel financially stressed, and to experience changes in their workload. Besides, all respondents experienced increased feelings of fear, anxiety, helplessness, and apprehension, mostly those into their 3rd trimester. Also, women in their 3rd trimester of pregnancy were certainly worried about the effect of the virus on themselves and their newborns, which can predispose them to prenatal depression [20]. In addition, this feeling of fear may be related to the approaching moment of the child’s birth, thus predisposing the pregnant woman to changes in her psychological well-being [31].

Family and social support also played an important role in our respondents’ mental well-being. In line with this, half of the respondents reported a positive relationship with their families, as evidenced by receiving increased support and sharing feelings with them, particularly those in their 1st and 3rd trimesters. In China, increased support from family members and friends during the early stages of the pandemic was reported by pregnant women in their 2nd and 3rd trimesters [20, 32]. In the current study, respondents also reported an unchanged level of care towards family members and sharing feelings with others when anxious, while in China pregnant women reported an increased level of care [20]. Our findings are consistent with previous studies carried out during the pre-COVID-19 era, which reported that social support alleviates anxiety among pregnant women [33, 34]. It is important to note that the respondents in this study abide by the Arab culture where family bonding is important and includes sharing emotions and strong affections between family members. In this culture, pregnancy is considered a joyful moment, where family members and friends show lots of affection and attention to the pregnant woman mostly before her delivery [35].

The pandemic induced mental health-related lifestyle changes among the respondents of this study, who showed increased time spent on resting and relaxing, as well as attention toward mental health well-being, regardless of the trimester of pregnancy. These findings were compatible with those reported by Zhang et al. [20]. On the other hand, time spent in exercise activities decreased as reported by half of the respondents due to the lockdown and closure of outdoor parks and fitness centers, and this differed significantly between trimesters [20]. Similarly, other studies reported decreased physical activity levels in pregnant women due to COVID-19 restrictions [36]. An important point to note is that women in the UAE are generally sedentary, especially during pregnancy [37]. However, the positive impact of physical activity on mental health (i.e., anxiety management and depression relief) is well-documented [38, 39] and also in regard to the benefits of being physically active during pregnancy [40].

Psychological stress impairs the immune system, rendering the human body more vulnerable to microbial infections [41]. In response, diets rich in vitamins A, C, D, B, E, iron, magnesium, zinc, copper, iodine, selenium, proteins, short-chain fatty acids, omega-3, polyphenols, probiotics have been highly emphasized lately to enhance the body’s immunity against COVID-19 [42, 43]. Also, dietary and herbal supplements are currently being promoted as “immune-boosters” in the treatment and prevention of COVID-19 [44, 45]. Interestingly, the majority of the pregnant women in this study did not consume any products to boost their immune system, possibly because they were worried about the safety of these products and potential risks to their unborn babies [46]. Respondents instead perceived that consumption of balanced diets, adequate fluids, and proper sleep are optimal measures to enhance their immunity during the pandemic. While the importance of exercise in relation to immunity was previously highlighted in a study conducted on Canadian pregnant women [47], it was not perceived by our respondents as one of the important immunity boosters.

In relation to eating behaviors, psychological and emotional distress are associated with unhealthy eating habits. Our respondents reported eating more homemade foods, with a marked decrease in fast food consumption and restaurant visits, which was surely related to the lockdown and closure of many dining places. Interestingly, “healthy food” options represented a small percentage of eating patterns pre-COVID times and dropped even further during the pandemic; this indicates that foods cooked at home were unhealthy with possibly high fat and/or sugar content. Our findings are compatible with the latest statistics released from Google Trends (by Google) in April 2020, in which a surge in searching for “recipes” and a decline in “healthy eating” were reported [48]. There was a rise in Google Trends searches for shelf-stable, processed, and energy-dense comfort foods (such as potato chips, popcorn, chocolate, and ice cream). In contrast, Spanish pregnant women experienced no change in eating behaviors and no episodes of overeating during the COVID-19 lockdown [36]. Predictive factors of mental well-being in our respondents included family and friends’ support, as well as taking immunity boosters. Similar findings were reported by Farewell et al. [49] regarding social support and healthy lifestyle habits (including healthy eating and physical activity) as important determinants of anxiety levels in pregnant and postpartum women. On the other hand, a study conducted in pregnant Japanese women reported that household finances and social support contributed significantly to higher IES-R scores [50].

Our findings suggest advocating more virtual activities to increase interactions between pregnant women, family members and friends, which may help in sustaining mental well-being during the pandemic. In addition, strategies to counter psychological distress and to boost immunity against the novel virus in pregnancy are also needed. The design of a bilingual online program (English and Arabic), with sessions on stress management and home-based healthy lifestyle tips, would be of great importance during such difficult times, especially for this group of people. The program should be adapted to the different socio-economic groups of varied nationalities within the UAE and include prenatal workout sessions.

The strengths of our study include a good representation of pregnant women from different Emirates and multi-centers in the UAE. In addition, we were able to capture the impact on mental health during the COVID-19 lockdown in the UAE. Our study measured different lifestyle-related factors that can be used in designing culturally adapted services by the local health authorities. Limitations to our study include that respondents’ recruitment was mainly conducted in the private sector among nationals of Arab countries (Emiratis and Arab expatriates), which could have influenced the results due to their particular sociodemographic characteristics. In addition, the history of psychiatric disorders was not collected, which could have influenced the severity of the mental health impact from COVID-19. Moreover, the lack of a pre-COVID-19 control group and a previous mental health assessment during pregnancy might limit the generalizability of the findings. The level and type of exercise were also not reported, which is an important influencer of gestational weight gain and overall women’s mental health and well-being. Future studies are needed to evaluate the long-term impact of COVID-19 on the mental health of expecting mothers and their mother-infant bonding. Qualitative and mixed research methods are recommended to provide a depth of understanding regarding the impact of the COVID-19 pandemic on pregnant women.

## Conclusion

The surge of COVID-19 cases remains a global threat to date. Pregnant women in the UAE are experiencing mild psychological distress and unhealthy lifestyle changes during the pandemic. The current study confirms that family support and sufficient time spent resting and relaxing are essential for the mental well-being of pregnant women. Given the known consequences of psychological distress on pregnancy and infant outcomes, there is a serious need for improving pregnant women’s mental and physical health during this stressful time.

## Data Availability

The datasets used and/or analysed during the current study are available from the corresponding author on reasonable request.

## Abbreviations

CI: Confidence Interval
COVID-19: Coronavirus 2019
IES-R: Impact of Event Scale-Revised
MHLSS: Mental Health Lifestyle Scale
PSS: Perceived Support Scale
PTSD: Post-Traumatic Stress Disorder
SD: Standard Deviation
SPSS: Statistical Package for the Social Sciences
UAE: United Arab Emirates
WHO: World Health Organization
